# CTAB-GAN+: enhancing tabular data synthesis

**DOI:** 10.3389/fdata.2023.1296508

**Published:** 2024-01-08

**Authors:** Zilong Zhao, Aditya Kunar, Robert Birke, Hiek Van der Scheer, Lydia Y. Chen

**Affiliations:** ^1^Faculty of Electrical Engineering, Mathematics and Computer Science, Delft University of Technology, Delft, Netherlands; ^2^Technical University of Munich School of Social Sciences and Technology, Technical University of Munich, Munich, Germany; ^3^Computer Science Department, University of Turin, Turin, Italy; ^4^AEGON, The Hague, Netherlands; ^5^Computer Science Department, University of Neuchâtel, Neuchâtel, Switzerland

**Keywords:** GAN, data synthesis, tabular data, differential privacy, imbalanced distribution

## Abstract

The usage of synthetic data is gaining momentum in part due to the unavailability of original data due to privacy and legal considerations and in part due to its utility as an augmentation to the authentic data. Generative adversarial networks (GANs), a paragon of generative models, initially for images and subsequently for tabular data, has contributed many of the state-of-the-art synthesizers. As GANs improve, the synthesized data increasingly resemble the real data risking to leak privacy. Differential privacy (DP) provides theoretical guarantees on privacy loss but degrades data utility. Striking the best trade-off remains yet a challenging research question. In this study, we propose CTAB-GAN+ a novel conditional tabular GAN. CTAB-GAN+ improves upon state-of-the-art by (i) adding downstream losses to conditional GAN for higher utility synthetic data in both classification and regression domains; (ii) using Wasserstein loss with gradient penalty for better training convergence; (iii) introducing novel encoders targeting mixed continuous-categorical variables and variables with unbalanced or skewed data; and (iv) training with DP stochastic gradient descent to impose strict privacy guarantees. We extensively evaluate CTAB-GAN+ on statistical similarity and machine learning utility against state-of-the-art tabular GANs. The results show that CTAB-GAN+ synthesizes privacy-preserving data with at least 21.9% higher machine learning utility (i.e., F1-Score) across multiple datasets and learning tasks under given privacy budget.

## 1 Introduction

In the modern business world, it is common for companies to leverage big data to gain valuable insights from a variety of internal and external sources. However, the in-depth knowledge contained within these data can often infringe upon personal privacy and lead to injustified analysis (Narayanan and Shmatikov, 2008). To mitigate the risk of data abuse and protect against privacy breaches, many governments introduced strict regulations such as GDPR (EU), CCPA&NYPA (US), and LGPD (Brazil), which enforces stringent data protection measures. This presents a challenge for data-driven industries as they must now seek out innovative, scientifically sound solutions that enable knowledge discoveries while adhering to the constraints of data privacy and government regulation.

One potential solution is the use of synthetic data. These synthetic data are not only statistically comparable to the original data but also exhibit the same utility in subsequent data analysis, and the artificial nature makes them compliant with GDPR. The generative adversarial network (GAN) (Goodfellow et al., 2014), which is composed of a generator and a discriminator, is an innovative generative model that has been proven effective in synthesizing images, and has recently been utilized to synthesize tabular data (Mottini et al., 2018; Park et al., 2018; Xu et al., 2019; Zhao et al., 2021). However, recent studies have shown that GANs may fall prey to membership inference attacks which greatly endanger the personal information present in the real training data (Chen et al., 2020b; Stadler et al., 2020). Therefore, it is imperative to safeguard the training of tabular GANs such that synthetic data can be generated without causing harm. To address these issues, prior studies (Jordon et al., 2018; Long et al., 2019; Torkzadehmahani et al., 2019; Torfi et al., 2020) rely on differential privacy (DP) (Dwork, 2008). DP is a mathematical framework that provides theoretical guarantees bounding the statistical difference between any resulting machine learning (ML) model trained with or without a particular individual's information in the original training dataset. Typically, this can be achieved by injecting calibrated statistical noise while updating the parameters of a network during back-propagation, i.e., DP stochastic gradient descent (DP-SGD) (Abadi et al., 2016; Xie et al., 2018; Chen et al., 2020a), or by injecting noise while aggregating teacher ensembles using the PATE framework (Papernot et al., 2016; Jordon et al., 2018).

Current state-of-the-art (SOTA) tabular GAN algorithms only consider two types of variables, namely, continuous and categorical, ignoring a significant class of mixed data type. It is also uncertain if existing solutions can effectively handle highly imbalanced or skewed variables. Furthermore, most SOTA DP GANs are evaluated on images, and their efficacy on tabular datasets needs to be verified. Existing DP GANs do not provide a well-defined consensus on which DP framework (i.e., DP-SGD or PATE) is optimal for training tabular GANs. Moreover, DP GAN algorithms such as Chen et al. (2020a) (GS-WGAN) and Jordon et al. (2018) (PATE-GAN) change the original GAN structure from one discriminator to multiple discriminators, which increases the complexity of the algorithm. Xie et al. (2018) (DP-WGAN) and Torfi et al. (2020) (RDP-GAN) use the weight clipping to bound gradients which introduces instability for GAN training.

In this study, we extend CTAB-GAN (Zhao et al., 2021) to a new algorithm CTAB-GAN+. The objectives of CTAB-GAN+ are two-folds: (1) further improve the synthetic data quality in terms of machine learning utility and statistical similarity; and (2) implement efficient DP into tabular GAN training to control its performance under different privacy budgets. To achieve the first goal, CTAB-GAN+ introduces a new feature encoder used for variables following single Gaussian distribution. Moreover, CTAB-GAN+ adopts the Wasserstein distance plus gradient penalty (hereinafter referred to as Was+GP) loss (Gulrajani et al., 2017) to further enhance the stability and effectiveness of GAN training. Finally, CTAB-GAN+ adds a new auxiliary model to improve the synthesis performance for regression tasks. To achieve the second goal, CTAB-GAN+ uses the DP-SGD algorithm to train a single instead of multiple discriminators as in PATE-GAN and GS-WGAN. This reduces the complexity of the algorithm. CTAB-GAN+ also applies DP-SGD to the generator and the auxiliary model when there are real data involved in the calculation of losses. Additionally, CTAB-GAN+ reduces the privacy cost by accounting for sub-sampling (Wang et al., 2019) of smaller subsets from the full dataset used to train the models.

We rigorously evaluate CTAB-GAN+ using two setups: (1) without DP to generate data as realistic as possible and (2) with DP under different privacy budgets to show the trade-off with data fidelity. Both setups rely on machine learning utility and statistical similarity of the synthetic data as evaluation metrics. Specifically, CTAB-GAN+ is tested on seven widely used machine learning datasets: Adult, Covertype, Credit, Intrusion, Loan, Insurance, and King against 9 SOTA tabular data generation algorithms: IT-GAN, CTGAN, TVAE, TableGAN, CWGAN, and MedGAN used in setup one, and PATE-GAN, DP-WGAN, and GS-WGAN used in setup two. In setup one, CTAB-GAN+ outperforms all baselines by at least 33.5% on accuracy and 56.4% on AUC. In setup two under the same privacy budget (i.e., ϵ = 1 and ϵ = 100), CTAB-GAN+ outperforms all SOTA DP GANs on average by at least 7.8% and 21.9% on F1-score.

The main contributions of this study can be summarized as follows: (1) Novel conditional adversarial network which introduces a classifier/regressor providing additional supervision to improve the utility for ML applications. (2) Efficient modeling of continuous, categorical, and mixed variables via novel data encoding. (3) Improved GAN training using well-designed information loss, downstream loss and generator loss along with Was+GP to enhance stability and effectiveness. (4) Constructed a simpler and more stable DP GAN algorithm for tabular data to control its performance under different privacy budgets. Our code is openly hosted at this github.^1^

## 2 Motivation

Through empirical analysis, we show how previous SOTA methods fall short in addressing challenges in industrial datasets. The specifics of our experimental setup are detailed in Section 5.1.

### 2.1 Single Gaussian variables

**Figure 1:**
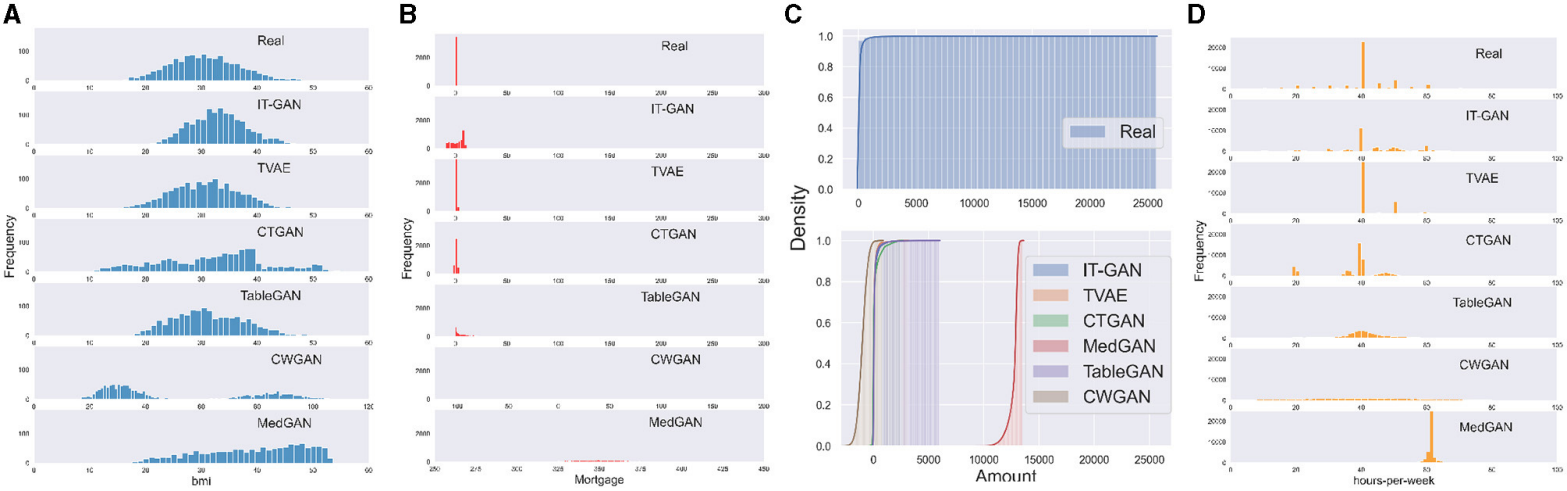
Challenges of modeling industrial dataset using existing GAN-based table generator: **(A)** single Gaussian, **(B)** mixed type, **(C)** long tail distribution, and **(D)** skewed data.

Single mode Gaussian distributions are very common. Figure 1A shows the histogram of variable *bmi* (i.e., body mass index) in the Insurance dataset, and synthetic data are generated by six SOTA algorithms for this variable. The distribution of real data is close to a single mode Gaussian distribution. But except TableGAN and TVAE, none of the SOTA algorithms can correctly recover this distribution in their synthetic data. IT-GAN reproduced the Gaussian distribution, but its mean and standard deviation shifted. CTGAN uses variational Gaussian mixture (VGM) to model all continuous variables. However, VGM is a complicated method to deal with single mode Gaussian distributions as it initially approximates the distribution with multiple Gaussian mixtures by default. TVAE also uses VGM to encode continuous column, but variational autoencoder (VAE) framework handles this use case better than GAN. CWGAN and MedGAN use min-max normalization to scale the original data to [0, 1]. TableGAN also uses min-max normalization but scales the original data to [−1, 1] to better match the output of the generator using *tanh* as activation function. The reason that min-max normalization works for TableGAN but not MedGAN and CWGAN is because the training convergence for both algorithms is less stable than for TableGAN. However, since TableGAN applies min-max normalization on all variables, it suffers from a disadvantage modeling column with complex multi-modal Gaussian distributions.

### 2.2 Mixed data type variables

As far as we are aware, existing GAN-based tabular data generators only recognize table columns as either categorical or continuous. However, in reality, a variable can possess qualities of both types and often exhibits missing values as well. A prime example of this is the *Mortgage* variable from the Loan dataset. Figure 1B displays the distribution of the original and synthetic data generated by six SOTA algorithms for this variable. As the data described, a loan holder can either have no mortgage (0 value) or a mortgage (any positive value). Despite the numerical nature of the data, all six SOTA algorithms treated the Mortgage variable as continuous, neglecting the unique significance of the value zero. As a result, all six algorithms generate values of approximately 0 rather than an exact 0. Furthermore, negative values for Mortgage hold no meaning in the real world.

### 2.3 Long tail distributions

Real-world data often exhibits long tail distributions, where the majority of occurrences are concentrated near the initial value of the distribution, with rare cases appearing toward the end. This can be seen in the cumulative frequency plots of data generated by six SOTA algorithms for the *Amount* variable in the Credit dataset, as shown in Figure 1C. This variable represents transaction amounts when using credit cards, and it is likely that the majority of transactions involve relatively small amounts, ranging from a few bucks to thousands. However, it is also possible for there to be a very small number of transactions with large amounts. It is noteworthy that for the purpose of comparison, both plots utilize the same x-axis, but the real data does not contain any negative values. The real data demonstrates that 99% of occurrences occur at the start of the range, with the distribution extending up to 25,000. In contrast, none of the synthetic data generators is able to effectively learn and replicate this behavior.

### 2.4 Skewed multi-mode continuous variables

The term *multi-mode* is derived from variational Gaussian Mixtures (VGM), which is discussed in more detail in Section 4.3. The rationale behind the use of multiple modes can be easily understood through the examination of Figure 1D. This figure plots the distribution of the working *hours-per-week* variable from the adult dataset, including the original data and synthetic data generated by all six SOTA algorithms. It is clear that the original distribution does not conform to a standard Gaussian shape. There is a prominent peak at 40 h, but there are also several lower peaks at 20, 45, and 50. Additionally, the altitude of approximately 20 is higher than those in 10 or 30. This type of behavior poses a challenge for SOTA data generators to capture. The closest results are obtained by IT-GAN and CTGAN which use VGM estimation for continuous variables. IT-GAN recovers most of the modes in the original distribution. However, the frequency of each mode changed, especially the dominant mode of approximately 40 h. CTGAN loses some modes compared to the original distribution.

The above examples show the shortcomings of current SOTA GAN-based tabular data generation algorithms and motivate the design of our proposed CTAB-GAN+.

## 3 Related work

The related study consists of two parts: (i) generative model for tabular data, and (ii) differential private tabular GANs.

### 3.1 Generative model for tabular data

Current state-of-the-art introduces several generative models for tabular data synthesis. MedGAN (Choi et al., 2017) combines a GAN with an autoencoder to synthesize electronic health record (EHR) data. While traditional GANs are only able to learn the distribution of continuous values, MedGAN's autoencoder is used to translate the continuous generation into categorical values. Mottini et al. (2018) adopted the Cramér Distance (Bellemare et al., 2017) and cross-net architecture (Wang et al., 2017) into the algorithm. In addition to generating with continuous and categorical data types, Mottini et al. (2018) also handled a missing value in the table by adding new variables. TableGAN (Park et al., 2018) includes an auxiliary classifier in the algorithm to improve the correlations between the generated features and labels for classification datasets. IT-GAN (Lee et al., 2021) uses neural ordinary differential equations (NODEs; Chen et al., 2018) as its generator. One advantage of this design is that IT-GAN can control the synthesis quality by controlling the negative log-density. However, the dimensionality of the generator's hidden layers cannot be changed, so an autoencoder is needed to compress the input into a fixed-length hidden representation. This design has the limitation that there is a loss of information during the compression from the table to the latent vector, which means that the GAN is unable to learn from the lost information.

In order to generate tabular data while conditioning on specific class of particular variable, conditional GAN is increasingly used. The ability to generate data for specific classes is important when the available data is limited and highly skewed as it allows the creation of synthetic data to rebalance the distribution. CWGAN (Engelmann and Lessmann, 2020) utilizes the Wasserstein distance (Arjovsky et al., 2017) in the conditional GAN framework and uses a conditional vector to oversample the minority class in order to address imbalanced tabular data generation. CTGAN (Xu et al., 2019) and TVAE (Xu et al., 2019) used a variational Gaussian mixture to encode continuous columns in order to handle complex data distributions. CTGAN also adopts a strategy called *training-by-sampling*, which leverages the use of the conditional vector to address imbalanced categorical variable problems.

CTAB-GAN+ combines the strengths of previous approaches, such as Was+GP, auxiliary classifier along with the effective encodings. Additionally, CTAB-GAN+ addresses the challenges of single Gaussian and long-tail variable distributions and introduces a new conditional vector structure to better handle imbalanced continuous variable distribution.

### 3.2 Differential private tabular GANs

To avoid leaking sensitive information on single individuals, previous studies explored multiple differential private learning techniques applied to GANs. Table 1 provides an overview. PATE-GAN (Jordon et al., 2018) uses PATE (Papernot et al., 2016) which relies on output sanitization by perturbing the output of an ensemble of teacher discriminators via Laplacian noise to train a student discriminator scoring the generated samples. One key limitation is that the student discriminator only sees synthetic data. Since these data are potentially unrealistic, and the provided feedback can be unreliable. (Xie et al., 2018) (DP-WGAN), (Chen et al., 2020a) (GS-WGAN), and (Torfi et al., 2020) (RDP-GAN) used differential private stochastic gradient descent (DP-SGD) coupled with the Wasserstein loss. Moreover, DP-WGAN uses a momentum accountant, whereas GS-WGAN and RDP-GAN used a Rényi Differential Privacy (RDP) accountant. The Wasserstein loss is known to be more effective against mode-collapse compared to KL divergence (Arjovsky et al., 2017). The RDP accountant provides tighter bounds on the privacy costs improving the privacy-utility trade-off. To incorporate differential privacy guarantees and make the training compatible with the Wasserstein Loss, Xie et al. (2018) and Torfi et al. (2020) used weight clipping to enforce the Lipschitz constraint. The drawback is the need for careful tuning of the clipping parameter (see Section 4.7). To overcome this issue, Chen et al. (2020a) enforced the Lipschitz constraint via a gradient penalty term as suggested by Gulrajani et al. (2017) but addressed only images and studies its efficacy only for training the generator network.

**Table 1:** Overview of DP GANs.

**Model**	**DP algo**	**Loss**	**WC**	**DP site**	**#D**	**Noise**	**Account**.	**Data format**
PATEGAN	PATE	KL Diver.	N	D	M	Lap.	PATE	Table
DP-WGAN	DP-SGD	Was.	Y	D	S	N	Moment	Image & Table
GS-WGAN	DP-SGD	Was. + GP	N	G	M	N	RDP	Image
RDP-GAN	DP-SGD	Was.	Y	D	S	N	RDP	Table
CTAB-GAN+	DP-SGD	Was. + GP	N (D, G), S (C)	G, D, C	S	N	RDP	Table

Diver, Divergence; Was, Wasserstein; WC, Weight Clipping; GP, Gradient Penalty; Lap, Laplacian noise; N, No; Y, Yes; N, Gaussian noise; S, Single; M, Multiple; G, Generator; D, Discriminator; C, Auxiliary model.

The proposed CTAB-GAN+ leverages RDP-based privacy accounting comparing to PATE used by PATE-GAN. Same as DP-WGAN, CTAB-GAN+ uses one discriminator instead of multiple ones trained by PATE-GAN and GS-WGAN. Since CTAB-GAN+ adopts Was+GP loss, it intrinsically constraints the gradient norm allowing to forgo the weight clipping used in DP-WGAN. This leads to a more stable training. In a nutshell, CTAB-GAN+ by training only one discriminator with Was+GP loss results in a more stable DP GAN algorithm compared to the SOTA algorithms.

## 4 CTAB-GAN+

In order to address the challenges described in Section 2, CTAB-GAN+ introduces several new features. One of these features is a redesigned min-max scaler that normalizes single Gaussian variable as well as a novel mixed-type encoder that can effectively represent mixed categorical-continuous variables and missing values. CTAB-GAN+ incorporates Was+GP, downstream, information, and generator losses (Gulrajani et al., 2017; Odena et al., 2017; Park et al., 2018; Xu et al., 2019) to improve synthetic data quality and training stability. Additionally, the newly designed conditional vector can counter the mode-collapse problem for both the imbalanced categorical and continuous variables. Finally, differential private SGD training is implemented for all the components to achieve strict privacy guarantees.

### 4.1 Technical background

#### 4.1.1 Tabular GAN

GANs have proven its utility for synthesizing tabular data in previous studies (Yahi et al., 2017; Park et al., 2018; Xu et al., 2019). There are many excellent methods that we can learn from.

Modeling imbalanced dataset can be a challenge for GANs as they can cause models to disproportionately favor the majority class. To address this issue, we adopted the *training-by-sampling* method from CTGAN. This approach involves the use of a conditional vector, which represents the classes of categorical columns. The vector is used to both feed the generator and discriminator and to sample subsets of the real training data that satisfy the given condition. By leveraging this condition, we can resample all classes and give minority classes a higher chance of being included in the training data.

To more effectively represent tabular data, we use one-hot encoding for all the categorical variables. To handle the complex distributions of continuous columns, we adopted the *Mode-Specific Normalization (MSN)* method (Xu et al., 2019). This involves encoding each value as a value-mode pair based on a variational Gaussian mixture model.

To improve the stability of GAN training, CTAB-GAN+ adopts the Was+GP (Gulrajani et al., 2017) loss, which was proposed to address the issues of exploding and vanishing gradients that can arise with the use of gradient clipping in the original WGAN (Arjovsky et al., 2017). Comparing to WGAN (Arjovsky et al., 2017), Was+GP replaces weight clipping with a constraint on the gradient norm of the discriminator, which helps to further stabilize the training process and reduces the need for hyper-parameter tuning. One notable difference between Was+GP and other GAN methods is that the discriminator is updated five times per mini-batch of data, while the generator is only updated once. This has implications for our differential privacy budget, which is described in more detail in Section 4.7.

To enhance the generation quality, we incorporated three extra terms into the loss function of the generator: information (Park et al., 2018), downstream [referred as classification loss in Odena et al. (2017) for classification problems], and generator loss (Xu et al., 2019). The information loss is used to minimize the discrepancy between statistics of the real data and the generated data, helping to produce data that is more statistically similar to the real data. The downstream loss requires adding to the GAN architecture an auxiliary model (classifier or regressor) in parallel to the discriminator. The auxiliary model produces predictions based on the generated features. The downstream loss measures the discrepancy between the synthesized and predicted values in downstream analysis, helping to improve the semantic integrity of synthetic records. For instance, in a health record dataset, the record (sex = male, disease = uterine cancer) would not be semantically correct, as men do not have a uterus, and such a record would not exist in the original data, but “male” and “uterine cancer” are existing in the sex and disease columns, respectively. The downstream loss, which is used by TableGAN for classification tasks, helps to prevent the generation of semantically incorrect records. CTAB-GAN+ extends the use of the downstream loss to regression datasets. The generator loss measures the difference between the specified conditions and the output classes of the generator. This loss helps the generator to learn to produce the exact same classes as the given conditions.

#### 4.1.2 Differential privacy

DP is becoming the standard solution for privacy protection and has even been adopted by the US census department to bolster privacy of citizens (Hawes, 2020). DP protects against privacy attacks by minimizing the influence of any individual data point based on a given privacy budget. In this study, we leverage the Rényi Differential Privacy (RDP) (Mironov, 2017) as it provides stricter bounds on the privacy budget. A randomized mechanism M is (λ, ϵ)-RDP with order λ and privacy budget ϵ if


(1) $$D_{\lambda }(ℳ(S)||ℳ(S^{\prime }))=\frac{1}{\lambda -1}logE_{x\sim ℳ(S)}{\left[\left(\frac{Pr[ℳ(S)=x]}{Pr[ℳ(S^{\prime })=x]}\right)\right]}^{\lambda -1}\leq \epsilon$$

holds for any adjacent datasets *S* and *S*′, where *Pr* denotes the probability density at given condition and $D_{\lambda }\left(P||Q\right)=\frac{1}{\lambda -1}logE_{x\sim Q}\left[{\left(P\left(x\right)/Q\left(x\right)\right)}^{\lambda }\right]$ represents the Rényi divergence for two probability distributions P and Q (Chen et al., 2020a). In addition, a (λ, ϵ)-RDP mechanism M can be expressed as


(2) $$\begin{array}{l} \left(\epsilon +\frac{log1/\delta }{\lambda -1},\delta \right)\text{-DP}. \end{array}$$

where δ denotes the probability of breaching DP constraints. For the purpose of this study, M corresponds to a tabular GAN model.

RDP is a strictly stronger privacy definition than DP as it provides tighter bounds for tracking the cumulative privacy loss over a sequence of mechanisms via the composition theorem (Mironov, 2017). Let ° denote the composition operator. For $M_{1}$,…,$M_{k}$ such that $M_{i}$ is (λ, ϵ_*i*_)-RDP ∀*i* , the composition $M_{1}$°…°$M_{k}$ is


(3) $$\begin{array}{l} \left(\lambda ,\underset{i}{\sum }\epsilon_{i}\right)\text{-RDP} \end{array}$$

Additionally, for a Gaussian mechanism (Dwork and Roth, 2014), $M_{\sigma }$ parameterized by σ as


(4) $$\begin{array}{l} M_{\sigma }\left(x\right)=f\left(x\right)+N\left(0,\sigma^{2}I\right) \end{array}$$

where *f* denotes an arbitrary function with sensitivity $\Delta_{2}f=\underset{S,S^{\prime }}{\max}||f\left(S\right)-f\left(S^{\prime }\right)|{|}_{2}$ over all adjacent datasets *S* and *S*′ and N represents a Gaussian distribution with zero mean and covariance σ^2^*I* (where *I* is the identity matrix), $M_{\sigma }$ satisfies $\left(\lambda ,\frac{\lambda \Delta_{2}f^{2}}{2\sigma^{2}}\right)$-RDP (Mironov, 2017).

Lastly, two more theorems are key to this study. The post-processing theorem (Dwork and Roth, 2014) states that if M satisfies (ϵ, δ)-DP, F○M will satisfy (ϵ, δ)-DP, where *F* can be any arbitrary randomized function. Hence, it suffices to train one of the two networks in the GAN architecture with DP guarantees to ensure that the overall GAN is compatible with differential privacy. RDP for subsampled mechanisms (Wang et al., 2019) computes the reduction in privacy budget when sub-sampling private data. Formally, let X be a dataset with *n* data points and **subsample** return *m* ≤ *n* subsamples without replacement from X (subsampling rate γ = *m*/*n*). For all integers λ≥2, if a randomized mechanism M is (λ, ϵ(λ))-RDP, then M○subsample is


(5) $$\begin{array}{l} \left(\lambda ,\epsilon^{\prime }\left(\lambda \right)\right)\text{-RDP} \end{array}$$

where


$$\begin{array}{l} \epsilon^{\prime }(\lambda )\leq \frac{1}{\lambda -1}log(1\\ \;+\gamma^{2}\left(\begin{array}{c} \lambda \\ 2 \end{array}\right)\min\left\{4(e^{\epsilon (2)}-1),e^{\epsilon (2)}\min\{2,{\left(e^{\epsilon (\infty )}-1\right)}^{2}\}\right\}\\ \;+\sum_{j=3}^{\lambda }\gamma^{j}\left(\begin{array}{c} \lambda \\ j \end{array}\right)e^{\left(j-1)\epsilon (j\right)}\min\{2,(e^{\epsilon (\infty )}-1)j)\}) \end{array}$$

According to (1), each fixed λ can be used as a privacy measure, Wang et al. (2019) emphasized its *function view* in which ϵ is a function of λ, and this function is fully determined by M. The function is denoted by ϵ(λ). When λ = ∞, it indicates that M is (ϵ, 0)-DP, i.e., pure DP.

### 4.2 Architecture of CTAB-GAN+

**Figure 2:**
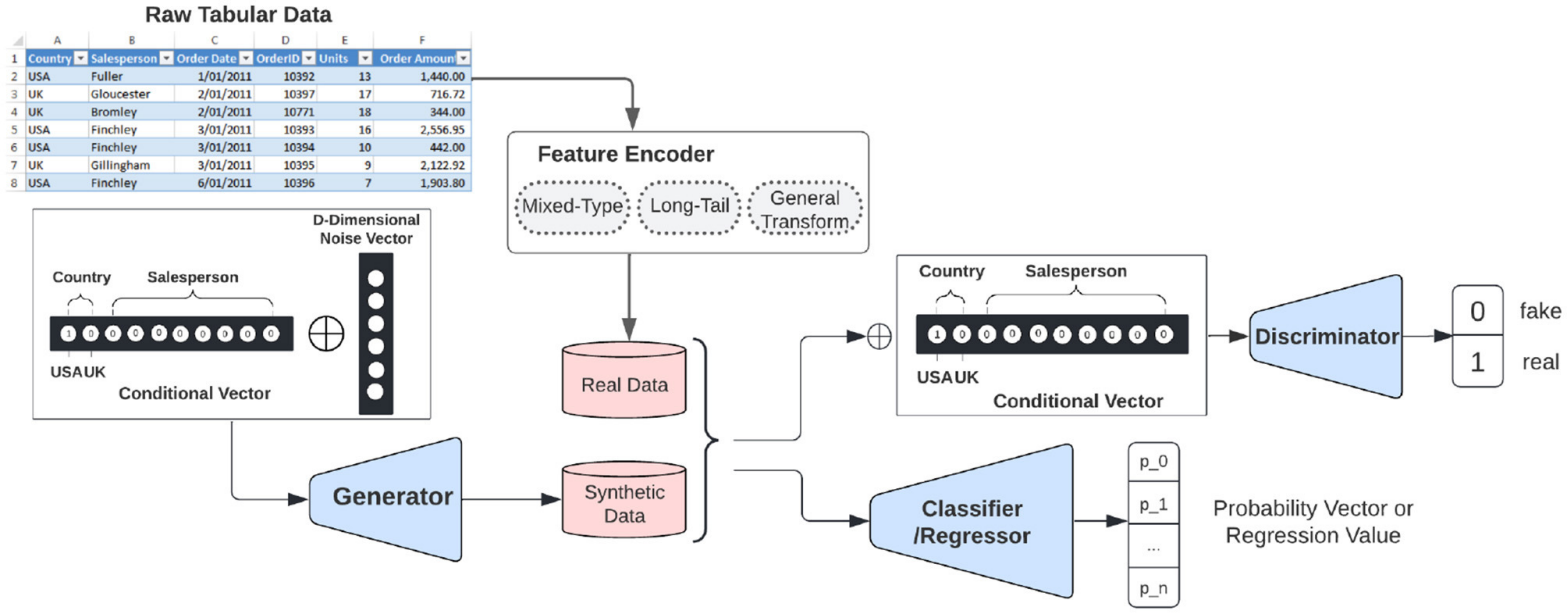
Synthetic tabular data generation via CTAB-GAN+.

The structure of CTAB-GAN+ is shown in Figure 2. It comprises three blocks: generator G, discriminator D, and an auxiliary component (either a classifier or a regressor) C. The input of G requires a noise vector plus a conditional vector. The conditional vector construction details are given in Section 4.4. Before feeding data to D and C, variables are encoded via different feature encoders depending on the variable type and characteristics. The details of the used encoders are provided in Sections 4.3, 4.5, and 4.6. The input of D needs to also concatenate the same conditional vector as given to G.

GANs are trained via a zero-sum min-max game where the goal of the generator being to produce synthetic data that is indistinguishable from real data, and the goal of the discriminator being to accurately distinguish between real and synthetic data. In the specific case described in the text, G is trained using additional feedback based on three loss terms: the information loss, the downstream loss, and the generator loss. The information loss measures the difference between the first- and second-order statistics (mean and standard deviation) of the synthetic and real data, encouraging the synthetic data to have the same statistical properties as the real data. The downstream loss measures the correlation between the target column and other columns in the data, ensuring that the combination of values in the synthetic data are semantically correct. The generator loss is the cross-entropy between the given conditional vector and the generated output classes, encouraging the generator to produce exactly the same output classes as the given conditional vector. These three loss terms are added to the default loss term (i.e., Was+GP) of G during training. We adopted the CNN structure from Park et al. (2018) for G and D. CNNs are good at capturing the relation between pixels within an image, which in our case, can help to increase the semantic integrity of synthetic data. The input data, which consists of row records stored as vectors, are processed by wrapping it in the closest square matrix dimensions and padding missing values with zeros. C, implemented using a multi-layer perceptron (MLP) with four 256-neuron hidden layers, is trained on the real data to better interpret the semantic integrity of the synthetic data. The synthetic data is reverse transformed from its matrix encoding to a vector (details in Section 4.3), while the real data are encoded (details in Sections 4.3, 4.6) before being used as input for C to create the class label predictions.

Suppose the last layer of D is softmax, then we used *f*_*x*_ and $f_{G\left(z\right)}$ that denote the logits fed into this softmax layer from a real sample *x* and a sample generated from latent value *z*, respectively. The **information loss** for G is calculated as


$$\begin{array}{l} {ℒ}_{info}^{G}=||E[f_{x}{]}_{x\sim p_{data}(x)}-E[f_{G(z)}{]}_{z\sim p(z)}|{|}_{2}\\ \;+||SD[f_{x}{]}_{x\sim p_{data}(x)}-SD[f_{G(z)}{]}_{z\sim p(z)}|{|}_{2} \end{array}$$

where *p*_*data*_(*x*) and *p*(*z*) denote prior distributions for real data and latent variable, 𝔼 and 𝕊𝔻 denote the mean and standard deviations of the features, respectively. The **downstream loss** is expressed as


$$\begin{array}{l} L_{dstream}^{G}=E{\left[\left|l\left(G\left(z\right)\right)-C\left(fe\left(G\left(z\right)\right)\right)\right|\right]}_{z\sim p\left(z\right)} \end{array}$$

where *l*(.) returns the target variable and *fe*(.) returns the input features of a given data record. Finally, the **generator loss** is presented as


$$\begin{array}{l} L_{generator}^{G}=H\left(m_{i},{\hat{m}}_{i}\right) \end{array}$$

where *m*_*i*_ and ${\hat{m}}_{i}$ are the given and generated conditional vector bits corresponding to column *i* and *H*(.) is the cross-entropy loss. Condition in column *i* is selected using the training-by-sampling procedure (see Section 4.4 for details).

Let $L_{default}^{D}$ and $L_{default}^{G}$ denote the GAN loss of discriminator and generator from Was+GP. Its unique objective function of discriminator is defined as follows:


$${ℒ}^{D}=\underset{\text{original discriminator loss}}{\underset{︸}{\underset{\tilde{x}\sim {\mathbb{P}}_{g}}{E}[D(\tilde{x})]-\underset{x\sim {\mathbb{P}}_{r}}{E}[D(x)]}}+\underset{\text{gradient penalty}}{\underset{︸}{\lambda \underset{\hat{x}\sim {\mathbb{P}}_{\hat{x}}}{E}{\left[({\left\|\nabla_{\hat{x}}D(\hat{x})\right\|}_{2}-1]\right)}^{2}}}$$

where ${\mathbb{P}}_{\hat{x}}$ is defined as sampling uniformly along straight lines between pairs of points sampled from the real data distribution ℙ_*r*_ and the generator distribution ℙ_*g*_. For G, the complete training objective is


$$\begin{array}{l} L^{G}=L_{default}^{G}+L_{info}^{G}+L_{dstream}^{G}+L_{generator}^{G} \end{array}$$

The training objective for D is unchanged. Finally, the loss to train the auxiliary C is similar to the downstream loss of the generator:


$$\begin{array}{l} L_{dstream}^{C}=E{\left[\left|l\left(x\right)-C\left(fe\left(x\right)\right)\right|\right]}_{x\sim p_{data}\left(x\right)} \end{array}$$

### 4.3 Mixed-type Encoder

**Figure 3:**
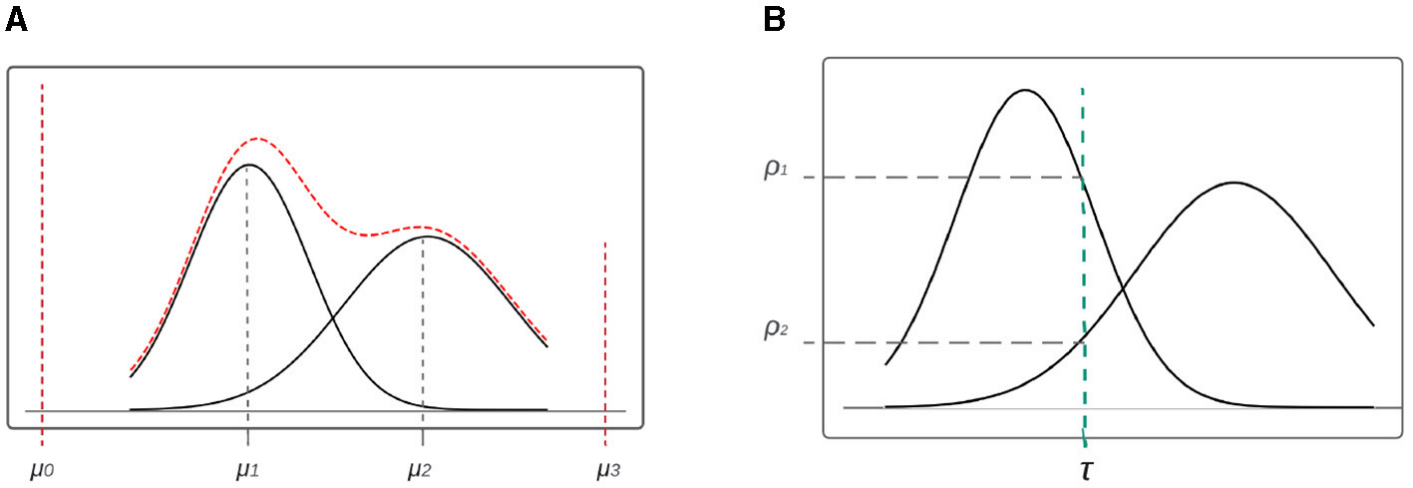
Encoding for mix data-type variable. **(A)** Mixed type variable distribution with VGM. **(B)** Mode selection of single value in continuous variable.

The tabular data are organized in rows and columns, and each column is encoded before it is used as input for training. We distinguished three types of variables: categorical, continuous, and mixed. Mixed columns contain both categorical and continuous values, or any column with missing values. To handle mixed columns, we propose a new mixed-type encoder that treats them as concatenated value-mode pairs. As an example, the encoding of a mixed variable is shown in red in Figure 3A. The values in this column can either be exactly μ_0_ or μ_3_ (the categorical part) or continuously distributed around two peaks in μ_1_ and μ_2_. To handle the continuous part, we adopted the *Mode-Specific Normalization* (MSN) idea from Xu et al. (2019) in using a variational Gaussian mixture (VGM) (Bishop, 2006) model to estimate the number of modes *k*, e.g., *k* = 2 in our example, and fit a Gaussian mixture. The learned Gaussian mixture is $\mathbb{P}=\overset{2}{\underset{k=1}{\sum }}\omega_{k}N\left(\mu_{k},\sigma_{k}\right)$, where N is the normal distribution, and ω_*k*_, μ_*k*_, and σ_*k*_ are the weight, mean, and standard deviation of each mode, respectively.

In order to encode values within the continuous region of the variable distribution, we establish a relationship between each value and the mode with the highest probability of occurrence, which is then normalized accordingly (see Figure 1B). To determine the appropriate mode, we first consider the probability density of both ρ_1_ and ρ_2_ for a given variable value τ. Once the mode with the highest probability is selected (in our example, mode 1 due to its higher ρ_1_), we then proceeded to normalize the value, resulting in the normalized value $\alpha =\frac{\tau -\mu_{1}}{4\sigma_{1}}$. To keep track of the mode used to encode τ, we utilize one-hot encoding, with the specific mode (e.g., mode 1) being represented as a binary vector (in our example, β = [0, 1, 0, 0]). Finally, we concatenate α and β, expressed as α⊕β, to obtain the final encoding.

For the categorical value (such as μ_0_ or μ_3_ in Figure 3A), the normalized value α is simply set to 0, as the category is determined solely by the one-hot encoding component. As an illustration, for a given value within μ_1_, the final encoding can be expressed as 0⊕[1, 0, 0, 0].

The process of encoding categorical variables involves the utilization of a one-hot vector, denoted by γ. In the event of missing values, these are treated as a distinct class and an additional bit is added to the one-hot vector to represent it accordingly. For a given row that possesses *N* variables, it is encoded by concatenation of the encoding of all variable values, i.e., either (α⊕β) for continuous and mixed variables or γ for categorical variables. Having *n* continuous/mixed variables and *m* categorical variables (*n*+*m* = *N*) the final encoding is


(6) $$\begin{array}{l} V=\overset{n}{\underset{i=1}{⊕}}\alpha_{i}⊕\beta_{i}\overset{N}{\underset{j=n+1}{⊕}}\gamma_{j} \end{array}$$

### 4.4 Conditional vector construction

**Figure 4:**
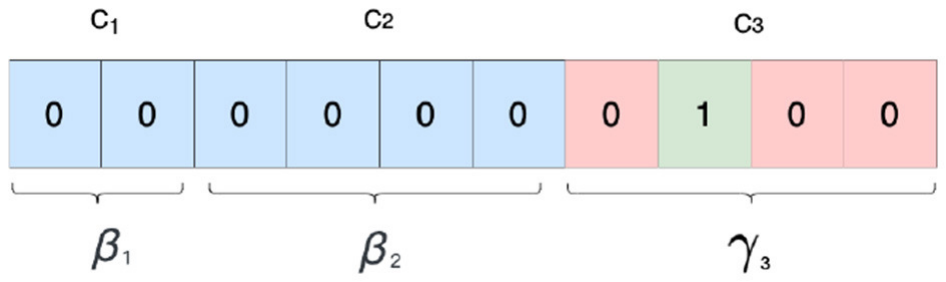
Conditional vector: example selects class 2 from third variable.

In CTAB-GAN+, we leverage conditional GAN to tackle the issue of imbalanced distribution in training datasets, extending the training-by-sampling approach (Xu et al., 2019). This method has been extended to incorporate the modes of continuous and mixed columns, thereby providing a more complete solution to the problem. When we sample real data, we used the conditional vector to filter and rebalance the training data. The conditional vector V is a bit vector given by the concatenation of all mode one-hot encodings β (for continuous and mixed variables) and all class one-hot encodings γ (for categorical variables) for all variables present in Equation 6. Each conditional vector specifies a single mode or a class, with V being a zero vector that has a single one in correspondence to the selected variable with the selected mode/class. Figure 4 shows an example with three variables, one continuous (*C*_1_), one mixed (*C*_2_), and one categorical (*C*_3_), with class 2 selected in *C*_3_.

In order to address the issue of imbalanced data distribution during training, we utilize a conditional vector which is constructed for each training iteration. To build this vector, we first randomly choose a variable with equal probability. We then estimate the probability distribution of each mode (or class for categorical variables) within the selected variable, using **logarithm** of frequency as a proxy. By using the logarithm of the probabilities instead of the original frequencies, we increase the chances of including rare modes/classes in the training process and prevent the issue of collapsing.

To rebalance the dataset, each time we need a conditional vector during training, we first randomly choose a variable with uniform probability. Then, we calculated the probability distribution of each mode (or class for categorical variables) in that variable using frequency as proxy and sample a mode based on the logarithm of its probability. Using the log probability instead of the original frequency gives minority modes/classes higher chances to appear during training. This helps to alleviate the collapse issue for rare modes/classes. Extending the conditional vector to include the continuous and mixed variables helps to deal with imbalance in the frequency of modes used to represent them.

### 4.5 General transform

CTAB-GAN originally adopts the mode-specific-normalization (MSN) from CTGAN to encode all continuous variables. MSN uses VGM to estimate the distribution of continuous variables. Figure 1A shows that VGM is not suitable for simple distributions such as single Gaussian. Another problem is the dimensionality explosion caused by using one-hot-encoding for categorical variables with a high number of categories. To counter both problems we propose the general transform (GT). GT is an effective approach to minimize the complexity of our algorithm.

The main idea of GT is to encode columns in the range of (−1, 1). This makes the encoding directly compatible with the output range of the generator using *tanh* activation function. This is achieved via a shifted and scaled min-max normalization. Mathematically, given a data point *x*_*i*_ of a continuous variable *x*, the transformed value, $x_{i}^{t}=2*\frac{x_{i}-min\left(x\right)}{max\left(x\right)-min\left(x\right)}-1$, where *min*(*x*) and *max*(*x*) represents the minimum and maximum values of the continuous variable. Inversely an encoded or generated value $x_{i}^{t}$ may be reverse transformed as $X_{i}=\left(max\left(x\right)-min\left(x\right)\right)*\frac{X_{i}^{t}+1}{2}+min\left(x\right)$. Continuous variable can be directly treated with the above formulas for normalization and denormalization. Categorical variables are first encoded using integers before using the above normalization and rounded to integers after using the above denormalization.

A similar transform was first introduced by TableGAN, but it applies this transformation on all variables. This choice is not optimal. From our experiments, we find that this technique only works well for continuous columns with simple distributions such as a single-mode Gaussian and does not cater to more complex distributions. By default, CTAB-GAN+ deals with continuous variable with MSN and only selectively uses GT for processing single-mode Gaussian variables. Similarly, when the categorical column is high dimensional and we decide to use another encoder instead of one-hot encoding, categorical columns should prefer MSN as encoding rather than GT. Using GT loses the mode indicator, i.e., β_1_ in Figure 4, from the conditional vector forgoing the ability to enhance the correlation between variables for specific categories. Moreover, using integers instead of one-hot vectors can impose artificial distances between the different categories which do not reflect the reality. Therefore, we recommend to use GT for categorical variables only if the categorical variables contain so many categories that the available machines can not train with the encoded data and the distribution of the categories is single-mode Gaussian.

### 4.6 Long tails treatment

We use variational Gaussian mixtures (VGM) to encode continuous values and handle multi-modal data distributions (as explained in Section 4.3). However, this method is not suitable for all types of data distributions, particularly those with long tails where a few rare points are significantly far from the majority of the data. Encoding values toward the tail of such distributions becomes challenging for VGM. To address this issue, we apply a logarithm transformation to pre-process variables with long tail distributions. For a given variable with a lower bound of *l*, we replace each value τ with τ^*c*^:


(7) $$\tau^{c}=\left\{\begin{array}{ll} \text{log}(\tau ) & \text{if }l>0\\ \text{log}(\tau -l+\epsilon ) & \text{if }l⩽0\text{, where }\epsilon >0 \end{array}\right\}$$

By applying the logarithmic transformation to columns with long tail distributions, we can reduce the distance between rare tail values and the bulk data. This can help VGM to encode all values more effectively. We demonstrate the effectiveness of this simple yet powerful technique in Section 5.6.

### 4.7 Differential privacy

DP-SGD (Abadi et al., 2016) is the central framework to provide DP guarantees in this study. DP-SGD uses noisy stochastic gradient descent to limit the influence of individual training samples *x*_*i*_. After computing the gradient *g*(*x*_*i*_), the gradient is clipped based on a clipping parameter *C* and its L2 norm $ḡ\left(x_{i}\right)\leftarrow g\left(x_{i}\right)/\max\left(1,\frac{||g\left(x_{i}\right)|{|}_{2}}{C}\right)$, and Gaussian noise is added $\tilde{g}(x_{i})\leftarrow \bar{g}(x_{i})+N(0,\sigma^{2}C^{2}I))$. $\tilde{g}$ is then used in place of *g* to update the network parameters as in traditional SGD.

One of the biggest challenges with DP-SGD is tuning the clipping parameter *C* since clipping greatly degrades the information stored in the original gradients (Chen et al., 2020a). Choosing an optimal clipping value that does not significantly impact utility is crucial. However, tuning the clipping parameter is laborious as the optimal value fluctuates depending on network hyperparameters (i.e., model architecture and learning rate) (Abadi et al., 2016). To avoid an intensive hyper-parameter search, Chen et al. (2020a) proposes to use the Wasserstein loss with a gradient penalty term for training discriminator in GANs. This term ensures that the discriminator generates bounded gradient norms which are close to 1 under real and generated distributions. Therefore, an optimal clipping threshold of *C* = 1 is obtained implicitly for DP-SGD on the discriminator and the generator. An empirical clipping value (Chen et al., 2020c) 1 is used for the auxiliary model.

When there is real data involved, CTAB-GAN+ trains all its components (i.e., discriminator, generator, and auxiliary model) using DP-SGD where the number of training iterations is determined based on the total privacy budget ϵ. Thus, to compute the number of iterations, the privacy budget spent for every iteration must be bounded and accumulated. For this purpose, we use the subsampled RDP analytical moments accountant technique. To elaborate the process of adding noise on gradients and calculating privacy cost in CTAB-GAN+, we show the theoretical analysis of discriminator in below as an example, the generator and the auxiliary model share the same process.

****Corollary** 1**. Each discriminator update satisfies (λ, 2*Bλ*/σ^2^)-RDP where *B* is the batch size.

**Proof** 1. Let *f* = *clip*(ḡ_*D*_, *C*) be the clipped gradient of the discriminator before adding noise. The sensitivity is derived via the triangle inequality:


(8) $$\begin{array}{l} \Delta_{2}f=\underset{S,S^{\prime }}{\max}||f\left(S\right)-f\left(S^{\prime }\right)|{|}_{2}\leq 2C \end{array}$$

Since *C* = 1 as a consequence of the Wasserstein loss with gradient penalty, and by using (4), the Gaussian mechanism used within the DP-SGD procedure denoted as $M_{\sigma }$ parameterized by noise scale σ may be represented as being (λ, 2λ/σ^2^)-RDP. Furthermore, each discriminator update for a batch of real data points {*x*_*i*_, .., *x*_*B*_} can be represented as


(9) $${\tilde{g}}_{D}=\frac{1}{B}\sum_{i=1}^{B}{ℳ}_{\sigma }({▽}_{\theta_{D}}{ℒ}^{D}(\theta_{D},x_{i}))$$

where ${\tilde{g}}_{D}$ and θ_*D*_ represent the perturbed gradients and the weights of the discriminator network, respectively. $L^{D}$ is the loss function of discriminator. (9) may be regarded as a composition of *B* Gaussian mechanisms and treated via (3). The privacy cost for a single gradient update step for the discriminator can be expressed as $\left(\lambda ,\overset{B}{\underset{i=1}{\sum }}2\lambda /\sigma^{2}\right)$ or equivalently (λ, 2*Bλ*/σ^2^).

Note that $M_{\sigma }$ is only applied for those gradients that are computed with respect to the real training dataset (Abadi et al., 2016; Zhang et al., 2018). Hence, the gradients computed with respect to the synthetic data or the gradient penalty term are left undisturbed for discriminator. For the generator, the DP-SGD is only applied on the gradients that are calculated by the **information loss**. There are no real data involved in the **generator loss**. The default GAN loss for generator $L_{default}^{G}$ and **downstream loss** are the post processing of already DP-protected discriminator and auxiliary model; therefore, there is no need to apply DP-SDG to the generator for these losses.

Next, to further amplify the privacy protection of CTAB-GAN+, we rely on (5) with sub-sampling rate γ = *B*/*N*, where *B* is the batch size and *N* is the size of the training dataset. Intuitively, subsampling adds another layer of randomness and enhances privacy by decreasing the chances of leaking information about particular individuals who are not included in any given subsample of the dataset.

## 5 Experimental analysis for data utility

To demonstrate the synthetic tabular data quality of the proposed CTAB-GAN+, we conducted experiments using seven commonly used machine learning datasets. We compared the results with those obtained using six SOTA tabular data generators as well as CTAB-GAN. Our evaluation criteria include measures of ML utility, statistical similarity to the real data, and ablation analyses to highlight the efficacy of the unique components of CTAB-GAN and CTAB-GAN+.

### 5.1 Experimental setup

#### 5.1.1 Datasets

All synthesizers are evaluated on seven commonly used machine learning datasets. Three of them **Adult**, **Covertype**, and **Intrusion** are from the UCI machine learning repository.^2^ **Credit** and **Loan** are from Kaggle.^3^ The above five tabular datasets are used for classification tasks where the target variable is categorical. To consider also regression tasks we include two more datasets, **Insurance** and **King** from Kaggle^4^ where the target variable is continuous.

Due to computing resource limitations, 50K rows of data are sampled randomly in a stratified manner with respect to the target variable for the Covertype, Credit, and Intrusion datasets. The Adult, Loan, Insurance and King datasets are taken in their entirety. The details of each dataset are shown in Table 2. We assume that the data type of each variable is known before training, a common assumption from previous studies (Xu et al., 2019; Lee et al., 2021).

**Table 2:** Datasets description.

**Dataset**	**Problem**	**Train/test split**	**Target variable**	**Continuous**	**Binary**	**Multi-class**	**Mixed-type**	**Long-tail**	**General transform**
Adult	Classification	39k/9k	“income”	3	2	7	2	0	1
Covertype	Classification	40k/10k	“Cover_Type”	10	44	1	0	0	47
Credit	Classification	40k/10k	“Class”	30	1	0	0	1	29
Intrusion	Classification	40k/10k	“Class”	22	6	14	0	2	6
Loan	Classification	4k/1k	“PersonalLoan”	5	5	2	1	0	9
Insurance	Regression	1k/300	“charges”	3	2	2	0	0	1
King	Regression	17.3k/4.3k	'price'	11	2	5	2	0	7

#### 5.1.2 Baselines

Our CTAB-GAN+ is compared with CTAB-GAN and six other SOTA tabular data generators: IT-GAN (Lee et al., 2021), CTGAN (Xu et al., 2019), TVAE (Xu et al., 2019), TableGAN (Park et al., 2018), CWGAN (Engelmann and Lessmann, 2020), and MedGAN (Choi et al., 2017). We implemented all algorithms in Pytorch and kept the hyperparameters, generator, and discriminator structures consistent with the descriptions provided in their studies. All the algorithms remain the same hyperparameters on all the datasets. For Gaussian mixture estimation of continuous variables, we set the default number of modes 10, the same as in CTGAN. We trained all algorithms for 150 epochs on Adult, Covertype, Credit, and Intrusion datasets. However, for Loan, Insurance, and King datasets, we trained all algorithms for 300 epochs as these datasets are smaller and require more training to converge. All experiments are repeated three times, and average results are reported.

#### 5.1.3 Environment

The experimental machine equips with an Intel i9 CPU with 10 cores, a GeForce RTX 2080 Ti GPU, and 32 GB of memory.

### 5.2 Evaluation metrics

The synthetic data are evaluated on two dimensions: (1) machine learning (ML) utility and (2) statistical similarity. They measure if the synthetic data can be used as a good proxy of the original data.

#### 5.2.1 Machine learning utility

**Figure 5:**
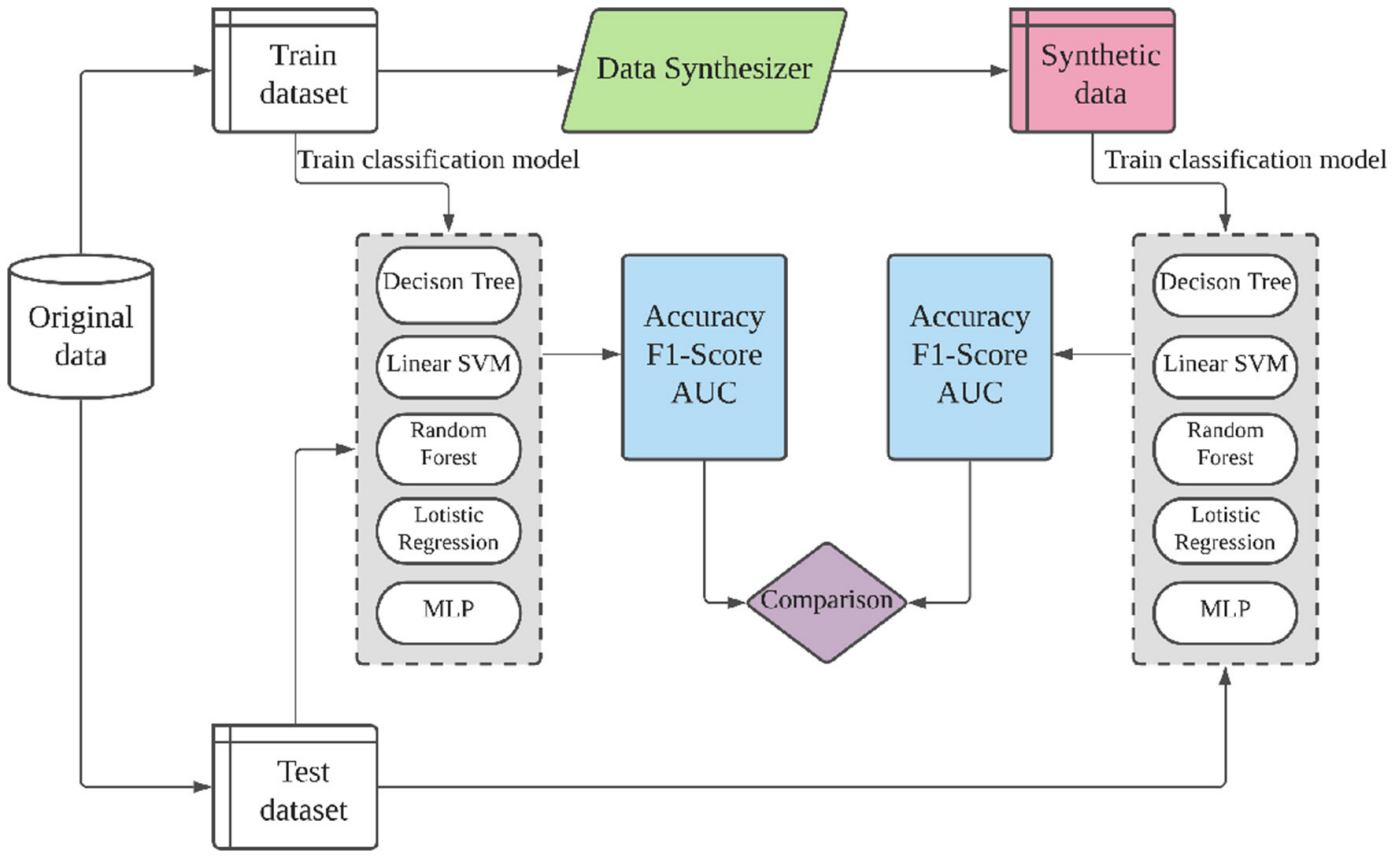
Evaluation flows for ML utility of classification.

The ML utility of classification and regression tasks is quantified differently. For classification, we quantify the ML utility via the performance, i.e, accuracy, F1-score, and area under the ROC curve (AUC), achieved by five widely used machine learning algorithms on real vs. synthetic data: decision tree classifier, linear support-vector-machine (SVM), random forest classifier, multinomial logistic regression, and MLP. Figure 5 shows the evaluation process for classification datasets. The training dataset and synthetic dataset are of the same size. The aim is to show the difference in ML utility when a ML model is trained on synthetic vs. real data. We used different classification performance metrics. Accuracy is the most commonly used but does not cope well with imbalanced target variables. F1-score and AUC are more stable metrics for such cases. AUC ranges from 0 to 1. For regression tasks, we quantify the ML utility in a similar manner but using four common regression algorithms—linear regression, ridge regression, lasso regression, and Bayesian ridge regression—and three regression metrics—mean absolute percentage error (MAPE), explained variance score (EVS), and *R*^2^ score. All algorithms are implemented using scikit-learn 0.24.2 with default parameters except max-depth 28 for decision tree and random forest, and 128 neurons for MLP. For a fair comparison, hyper-parameters are fixed across all datasets. Due to this, our results can slightly differ from Xu et al. (2019), where the authors use different ML models and hyper-parameters for different datasets.

#### 5.2.2 Statistical similarity

Three metrics are used to quantify the statistical similarity between real and synthetic data.

##### 5.2.2.1 Average Jensen-Shannon divergence (JSD)

The JSD is a measure of the difference between the probability mass distributions of individual categorical columns in the real and synthetic datasets. It is bounded between 0 and 1 and is symmetric, making it easy to interpret. We average the JSDs from all the categorical columns to obtain a compact, comprehensible score.

##### 5.2.2.2 Average Wasserstein distance (WD)

To measure the similarity between the distributions of continuous/mixed columns in synthetic and real datasets, we use the Wasserstein distance. We found that the JSD metric was numerically unstable for evaluating the quality of continuous columns, especially when there is no overlap between the synthetic and original datasets, so we chose to use the more stable Wasserstein distance instead. To make the WD scores comparable across columns, before computing the WD we fit and apply a min-max normalizer to each continuous column in the real data and apply the same normalizer to the corresponding columns in the synthetic data. We average all column WD scores to obtain the final score.

##### 5.2.2.3 Difference in pair-wise correlation (Diff. Corr.)

To evaluate the preservation of feature interactions in synthetic datasets, we compute the pair-wise correlation matrix separately for real and synthetic datasets. *Pearson correlation* coefficient is used between any two continuous variables. Similarly, the *Theil uncertainty* coefficient is used to measure the correlation between any two categorical features. The *correlation ratio* between categorical and continuous variables is used. Note that the dython^5^ library is used to compute these metrics. Finally, the difference between pair-wise correlation matrices for real and synthetic datasets is computed.

### 5.3 Results analysis

We first discuss the results in ML utility before addressing statistical similarity.

#### 5.3.1 ML utility

Table 3 shows the results for the classification datasets. A better synthetic dataset is expected to have small differences in ML utility for classification tasks trained on real and synthetic data. It can be seen that CTAB-GAN+ outperforms all other SOTA methods and CTAB-GAN in all the metrics. CTAB-GAN+ decreases the AUC difference from 0.094 (i.e., best baseline CTAB-GAN) to 0.041 (56.4% reduction), and the difference in accuracy from 7.86% (i.e., best baseline IT-GAN) to 5.23% (33.5% reduction). The improvement over CTAB-GAN shows that general transform encoder and Was+GP loss indeed help enhance the feature representation and GAN training. Table 4 shows the results for the regression datasets. The result of CTAB-GAN and CTAB-GAN+ are far better than all other baselines. This shows the effectiveness of the feature engineering. Additionally, as CTAB-GAN+ adds the auxiliary regressor which explicitly enhances the regression analysis, the overall downstream performance of CTAB-GAN+ is better than CTAB-GAN. We note that CTAB-GAN uses auxiliary classification loss for the classification analysis and disables it for the regression analysis.

**Table 3:** Difference (± standard deviation) of ML utility and statistical similarity for Classification between original and synthetic data, averaged on five datasets.

**Method**	**ML utility difference**			**Statistical similarity difference**		
	**Accuracy (%)**	**F1-score**	**AUC**	**Avg JSD**	**Avg WD**	**Diff. corr**.
CTAB-GAN+	**5.23 ± 1.493**	**0.090** **±** **0.009**	**0.041** **±** **0.003**	**0.039** **±** **0.002**	**0.010** **±** **0.001**	**2.03** **±** **0.039**
CTAB-GAN	8.90 ± 1.841	0.107 ± 0.008	0.094 ± 0.004	0.062 ± 0.002	0.013 ± 0.002	2.09 ± 0.031
IT-GAN	8.95 ± 1.911	0.229 ± 0.007	0.183 ± 0.002	0.078 ± 0.001	0.026 ± 0.002	2.63 ± 0.053
TVAE	7.86 ± 2.034	0.181 ± 0.010	0.140 ± 0.004	0.097 ± 0.002	0.017 ± 0.002	2.41 ± 0.055
CTGAN	21.51 ± 3.525	0.274 ± 0.012	0.253 ± 0.006	0.070 ± 0.002	0.025 ± 0.002	2.73 ± 0.097
TableGAN	11.40 ± 2.381	0.130 ± 0.009	0.169 ± 0.004	0.080 ± 0.001	0.055 ± 0.004	2.30 ± 0.078
MedGAN	14.11 ± 4.431	0.282 ± 0.017	0.285 ± 0.006	0.110 ± 0.004	0.159 ± 0.003	2.77 ± 0.181
CWGAN	20.06 ± 4.014	0.354 ± 0.022	0.299 ± 0.006	0.132 ± 0.002	0.136 ± 0.002	2.82 ± 0.167

A lower value indicates a better result. Best result in each column is highlighted in bold.

**Table 4:** Difference (± standard deviation) of ML utility and statistical similarity for regression between original and synthetic data, averaged on two datasets.

**Method**	**ML utility difference**			**Statistical similarity difference**		
	**MAPE**	**EVS**	*R* ^2^	**Avg JSD**	**Avg WD**	**Diff. corr**.
CTAB-GAN+	**0.04** **±** **0.011**	**0.03** **±** **0.014**	**0.04** **±** **0.002**	**0.040** **±** **0.001**	**0.014** **±** **0.001**	**0.65** **±** **0.044**
CTAB-GAN	0.06 ± 0.010	0.05 ± 0.006	0.06 ± 0.002	0.119 ± 0.003	0.042 ± 0.001	1.23 ± 0.040
IT-GAN	0.61 ± 0.025	0.41 ± 0.019	0.48 ± 0.003	0.097 ± 0.001	0.029 ± 0.001	2.23 ± 0.076
TVAE	0.24 ± 0.019	0.08 ± 0.009	0.22 ± 0.002	0.184 ± 0.002	0.021 ± 0.002	1.13 ± 0.058
CTGAN	0.87 ± 0.091	0.59 ± 0.047	0.71 ± 0.002	0.139 ± 0.002	0.035 ± 0.001	2.60 ± 0.117
TableGAN	0.34 ± 0.010	0.43 ± 0.006	0.48 ± 0.001	0.212 ± 0.003	0.031 ± 0.002	2.26 ± 0.039
MedGAN	0.98 ± 0.131	0.65 ± 0.172	0.43 ± 0.005	0.269 ± 0.004	0.145 ± 0.005	2.82 ± 0.175
CWGAN	0.64 ± 0.099	0.72 ± 0.073	0.46 ± 0.003	0.292 ± 0.003	0.159 ± 0.005	2.79 ± 0.076

A lower value indicates a better result.

#### 5.3.2 Statistical similarity

Statistical similarity results for the classification datasets are reported in Table 3 and for regression datasets in Table 4. CTAB-GAN+ stands out again across all baselines in both groups of datasets. For classification datasets, CTAB-GAN+ outperforms CTAB-GAN, CTGAN, and IT-GAN by 37.1%, 44.3%, and 50.0% in average JSD. This is due to the use of the conditional vector, the log-frequency sampling and the extra losses, which work well for both balanced and imbalanced distributions. CTAB-GAN+ also outperforms all the baselines for continuous variables. Comparing to CTAB-GAN, the significant improvement comes from the use of general transform to model continuous columns with simple distributions which originally used MSN under CTAB-GAN and CTGAN. For regression datasets, CTAB-GAN+ outperforms CTAB-GAN by 63.4 and 74.5% in average JSD and average WD, respectively. In addition to JSD and WD, the synthetic regression datasets maintain much better correlation than all the comparisons. This result confirms the efficacy of the usage of the auxiliary regressor.

### 5.4 Ablation analysis

Due to the page limit, ablation analysis are only implemented for classification datasets. We focus on conducting an ablation study to analyse the impact of the different components of CTAB-GAN and CTAB-GAN+.

#### 5.4.1 With CTAB-GAN

To assess the effectiveness of each strategy, we conducted four ablation studies that gradually remove components of CTAB-GAN. The experiments include (1) **w/o** C, where we remove C and the corresponding classification loss for Generator G from CTAB-GAN; (2) **w/o I. loss** (information loss), where we remove information loss from CTAB-GAN; (3) **w/o MSN**, where we use min-max normalization to replace MSN for continuous variables. Here, the conditional vector is the same as for CTGAN; and (4) **w/o LT** (long tail), where we exclude the long tail treatment. This only affects datasets with long-tailed columns, namely, Credit and Intrusion.

The results are compared with the default CTAB-GAN. Table 5 shows the results in terms of F1-score difference between ablation and CTAB-GAN on all five classification datasets. Each part of CTAB-GAN has different impacts on different datasets. For instance, **w/o** C has a negative impact for all datasets except Credit, where the small number of categorical variables limits the effectiveness of the semantic check. **w/o information loss** has a positive impact on Loan but leads to worse results for all other datasets. It can even make the model unusable for Intrusion. **w/o MSN** performs bad for Covertype but has little impact for Intrusion. Credit without MSN performs better than original CTAB-GAN. This is because 28 out of its 30 continuous variables are nearly single mode Gaussian distributed. The initialized high number of modes, i.e., 10, for each continuous variable (same setting as in CTGAN) degrades the estimation quality. **w/o LT** has the biggest impact on Intrusion since it contains two long tail columns which are important predictors for the target column. For Credit, the influence is limited. Even if the long tail treatment fits well the *amount* column (see Section 5.6), it is not a strong predictor for the target column.

**Table 5:** Ablation analysis for CTAB-GAN (F1. diff.).

**Dataset**	**CTAB-GAN**	**w/o C**	**w/o I. Loss**	**w/o MSN**	**w/o LT**
Adult	0.704	−0.01	−0.037	−0.05	-
Covertype	0.532	−0.018	−0.184	−0.118	-
Credit	0.710	+0.011	−0.177	+0.06	+0.001
Intrusion	0.842	−0.031	−0.437	+0.003	−0.074
Loan	0.803	−0.044	+0.028	+0.013	-

#### 5.4.2 With CTAB-GAN+

To show the efficacy of the General Transform and Was+GP loss in CTAB-GAN+, we propose two ablation studies. (1) **w/o GT** which disables the general transform in CTAB-GAN+. All continuous variables use MSN and all the categorical variables use one-hot encoding. (2) **w/o Was+GP** which switches the default GAN training loss from Was+GP to the original GAN loss defined in Goodfellow et al. (2014). It is worth noting that the information, downstream and generator losses are still present in this experiment. The other experimental settings are the same as in Section 5.4.1. Table 6 shows the results in terms of F1-score difference among different versions of CTAB-GAN+. For Covertype, Credit, and Intrusion datasets, the effects of GT and Was+GP are all positive. GT significantly boosts the performance on Covertype and Credit datasets. For Adult, it worsens the result. The reason is that the Adult dataset contains only one GT column: age. Since this column is strongly correlated with other columns, the original MSN encoding can better capture this interdependence. The positive impact of Was+GP on the other hand is limited but consistent across all datasets. The only exception is the Loan dataset, where GT and Was+GP have minor impacts. This is due to the fact that Loan has fewer variables comparing to other datasets, which makes it easier to capture the correlation between columns. CTAB-GAN already performs well on Loan, Therefore, GT and Was+GP cannot further improve performance on this dataset.

**Table 6:** Ablation analysis for CTAB-GAN+ (F1. diff.).

**Dataset**	**CTAB-GAN+**	**w/o GT**	**w/o Was+GP**
Adult	0.684	+0.013	−0.029
Covertype	0.636	−0.196	−0.012
Credit	0.802	−0.303	−0.08
Intrusion	0.912	−0.041	−0.049
Loan	0.806	−0.001	+0.003

### 5.5 Training time analysis

Previous results show that CTAB-GAN+ outperforms the SOTA in ML utility and statistical similarity. This comes at the expense of additional training complexity, i.e., Was+GP, information, downstream and generator losses, and feature encoding, i.e., mixed-type, long tail, and VGM mode in conditional vectors. To capture the additional costs, Table 7 shows the average training time per epoch for all models on all classification datasets. IT-GAN is the slowest because it trains an autoencoder on top of GAN. Since the dimensionality of its generator is unchanged for all the datasets, its training time is similar across datasets except on Loan due to its small size. Except IT-GAN, CTAB-GAN+ is slower than other algorithms in most cases. CTAB-GAN+ runs faster than CTAB-GAN on Credit and Loan datasets because of the usage of GT. For other datasets, CTAB-GAN+ uses more training time than CTAB-GAN per epoch due to the nature of Was+GP. Was+GP demands updating five times the discriminator per data batch as compared to a single update required by the original GAN loss. Actually, CTGAN and CWGAN also use Was+GP loss in their training. But with all the extra losses and auxiliary component, CTAB-GAN+ and CTAB-GAN are slower than other algorithms.

**Table 7:** Training time (s/epoch) usage.

**Dataset**	**CTAB.+**	**CTAB**.	**IT**.	**TVAE**	**CT**.	**Table**.	**Med**.	**CW**.
Adult	11.11	7.50	12.69	1.91	1.82	0.52	0.33	4.73
Covertype	9.24	8.38	12.74	4.09	3.44	1.69	0.33	10.41
Credit	3.79	8.92	11.51	10.46	2.46	1.73	0.34	4.50
Intrusion	12.54	10.73	12.83	6.90	3.09	1.75	0.34	10.81
Loan	0.44	0.57	1.19	0.29	0.18	0.06	0.04	1.12

To further improve the training efficiency of CTAB-GAN+, it is possible to pre-train the classifier/regressor with the real dataset before-hand instead of in parallel to the generator. Thus, by computing only the inference, we can speed up the calculation of the classifier loss. However, if we use that solution to address this issue, it may create another problem. This is because (Weng, 2019) suggests that when training the GAN with a perfect discriminator, the gradient of the loss function drops close to zero and learning stagnates. That is why we do not pre-train the discriminator but train it along side the generator. In similar vein, a well-trained classifier/regressor may not help the training of generator at least at the beginning.

### 5.6 Results for motivation cases

After reviewing all the metrics, let us recall the four motivation cases from Section 2.

#### 5.6.1 Single Gaussian variables

**Figure 6:**
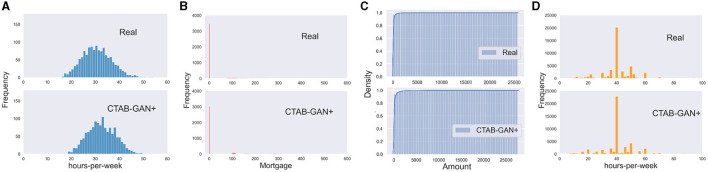
Modeling industrial dataset using CTAB-GAN+: **(A)** simple Gaussian, **(B)** mixed type, **(C)** long tail distribution, and **(D)** skewed data.

Figure 6A shows the real and CTAB-GAN+ generated *bmi* variable. CTAB-GAN+ can reproduce the distribution with minor differences. This shows the effctiveness of general transform to better model variables with single Gaussian distribution.

#### 5.6.2 Mixed data type variables

Figure 6B compares the real and CTAB-GAN+ generated variable *Mortgage* in Loan dataset. This variable is encoded as mixed type. We can see that CTAB-GAN+ generates clear “0” values and the frequency is close to real data.

#### 5.6.3 Long tail distributions

In Figure 6C, we present the cumulative frequency graph for the *Amount* column in the Credit dataset, which has a typical long-tail distribution. It can be observed that CTAB-GAN+ is able to recover the real distribution with high fidelity. This is mainly attributed to the log-transform data pre-processing used by CTAB-GAN+, which enables it to learn this complex structure significantly better than SOTA methods shown in Figure 1C.

#### 5.6.4 Skewed multi-mode continuous variables

Figure 6 shows the original *Hours-per-week* variable distribution from the adult dataset and the corresponding synthetic data distribution from CTAB-GAN+. The synthetic data do not only capture the dominant peak round 40 but also capture well other side peaks with lower frequency. The reason is that CTAB-GAN+ incorporates modes of VGM into conditional vector, and then we can apply the training-by-sample and logarithm frequency also to the modes of continuous variables. This gives the mode with less weight more chance to appear in the training and avoids the mode collapse.

### 5.7 Further discussion

The preceding findings highlight the efficacy of CTAB-GAN+ in modeling various types of data distributions, contributing to enhanced fidelity for synthetic data. However, this section delves into the limitations inherent in the current implementation of CTAB-GAN+.

The primary challenge lies in handling high-dimensional data during training. The use of one-hot encoding for categorical columns, such as ZIP-Code, poses a significant hurdle. In such cases, the encoded data becomes excessively lengthy and sparse, amplifying the difficulty for the model to discern data patterns. Additionally, extremely high-dimensional data may exceed the memory capacity of the training machine. While labeling each category with a numerical value and treating it as a continuous column can mitigate this issue, it introduces implicit distances between categories, deviating from the desired representation.

A second potential limitation lies in the permutation effect on synthetic data quality when altering the tabular data column order. Typically, permuting the column order of tabular data has no influence on its semantic meaning. However, this can potentially result in changes to the quality of synthetic data generated. During the feature encoding step, CTAB-GAN+ initially transforms each data row into an image and employs a convolutional neural network (CNN) for both the generator and discriminator functions to handle the data. CNNs are known for their proficiency in capturing regional feature relations. As demonstrated in the results presented in Tables 3, 4, CNN-based algorithms such as CTAB-GAN and CTAB-GAN+ surpass the performance of the fully-connected network-based CTGAN. Nevertheless, when a user permutes the column order, regional features undergo changes, leading to potential discrepancies in the information captured by the CNN and, consequently, variations in synthetic data quality.

Addressing these challenges necessitates the exploration of new feature engineering approaches and the development of novel model structures. These avenues remain open for future research endeavors.

## 6 Experiment analysis for differential privacy

In this section, we show the effect of activating DP in CTAB-GAN+ and compare CTAB-GAN+ with three SOTA DP GAN algorithms.

### 6.1 Experiment setup

#### 6.1.1 Datasets

Due to page limit, we only use the classification datasets: Adult, Covertype, Intrusion, Credit, and Loan.

#### 6.1.2 Metrics

We use the same ML utility metrics from Section 5.2 under two privacy budgets, i.e., ϵ = 1 and ϵ = 100.

#### 6.1.3 Baselines

CTAB-GAN+ is compared against three SOTA architectures: PATE-GAN (Jordon et al., 2018), DP-WGAN (Xie et al., 2018), and GS-WGAN (Chen et al., 2020a). The code of PATE-GAN and DP-WGAN is taken from the private data generation toolbox^6^ which already adapts them for tabular data synthesis. We extend GS-WGAN to the tabular domain by converting each data row into a bitmap image. We first normalized all values to the range [0, 1] and re-shaped rows in the form of square images filling missing entries (if any) with zeros. The re-shaped rows are fed into the algorithm, and the generated images are transformed into data rows by reversing the previous two operations. All hyper-parameters are kept to their default values except for the default network architecture which is adjusted according to the spatial dimensions of the tabular datasets. Lastly, note that to compute privacy cost fairly, the RDP accountant is used for all approaches that use DP-SGD as it provides tighter privacy guarantees than the moment accountant (Wang et al., 2019).

#### 6.1.4 Privacy accounting

To compute the privacy cost in a fair manner, we used the RDP accountant for all approaches that employ DP-SGD: CTAB-GAN+, DP-WGAN, and GS-WGAN. PATE-GAN uses moment accountant (Wang et al., 2019) by default. We set δ = 10^−5^ for all experiments. We follow the examples of DP-WGAN and set the exploration span of λ to [2, 4096]. We use (2) to convert the overall cumulative privacy cost computed in terms of RDP back to (ϵ, δ)-DP.

### 6.2 Results analysis

#### 6.2.1 ML utility

Table 8 presents the results for the differences ML utility between models trained on the original and synthetic data: lower is better. CTAB-GAN+ outperforms all other SOTA algorithms under both privacy budgets. With a looser privacy budget, i.e., higher ϵ, all metrics for all algorithms improve. These results are in line with our expectation because higher privacy budgets mean training the model with less injected noise and more training epochs—before exhaustion of the privacy budget. CTAB-GAN+ outperforms second best 7.8% in F1-Score under ϵ = 1, and this advantage increases to 21.9% when ϵ = 100. The superior performance of CTAB-GAN+ compared to other baselines can be explained by its well-designed neural network architecture, which improve the training objective and capacity to better deal with the challenges of the tabular domain such as column dependencies. This also explains the poor results offered by GS-WGAN which is not designed to handling these specific issues achieving the worst overall performance.

**Table 8:** Difference of accuracy (%), F1-score, AUC, and AP between original and synthetic data: average over 5 ML models and five datasets with different privacy budgets ϵ = 1 and ϵ = 100.

**Method**	ϵ = 1			ϵ = 100		
	**Accuracy (%)**	**F1-score**	**AUC**	**Accuracy (%)**	**F1-score**	**AUC**
CTAB-GAN+	**32.21**	**0.473**	**0.409**	**22.04**	**0.392**	**0.348**
PATE-GAN	38.17	0.513	0.447	37.96	0.508	0.394
DP-WGAN	36.88	0.536	0.510	27.27	0.502	0.423
GS-WGAN	64.10	0.668	0.495	58.22	0.639	0.488

A lower value indicates a better result. Best result in each column is highlighted in bold.

#### 6.2.2 Statistical similarity

Table 9 summarizes the statistical similarity results. Among all DP models, CTAB-GAN+ and GS-WGAN consistently improve across all metrics when the privacy budget is increased. But the performance of GS-WGAN is significantly worse than CTAB-GAN+. PATE-GAN and DP-WGAN also see improvements on Diff. Corr., which means the algorithms capture better column dependencies with higher privacy budgets. But there are no significant improvement on Avg JSD and Avg WD. This highlights the inability of this methods to capture the statistical distributions during training despite a looser privacy budget. This can be explained by the lack of effective feature engineering for dealing with complex statistical distributions present in the tabular domain which arise from imbalances in categorical columns and skews in continuous columns commonly found in real-world tabular datasets.

**Table 9:** Statistical similarity metrics between original and synthetic data: average on five datasets with different privacy budgets ϵ = 1 and ϵ = 100.

**Method**	ϵ = 1			ϵ = 100		
	**Avg JSD**	**Avg WD**	**Diff. corr**.	**Avg JSD**	**Avg WD**	**Diff. corr**.
CTAB-GAN+	**0.322**	**0.136**	**5.16**	**0.302**	**0.131**	**5.03**
PATE-GAN	0.356	0.221	9.45	0.366	0.221	8.94
DP-WGAN	0.362	0.221	9.19	0.359	0.221	9.49
GS-WGAN	0.624	0.472	15.61	0.547	0.397	10.90

A lower value indicates a better result. Best result in each column is highlighted in bold.

## 7 Conclusion

In this study, we propose CTAB-GAN+, a conditional GAN based tabular data generator. CTAB-GAN+ advances beyond SOTA methods by improving performance on regression datasets and allowing control over the quality of synthesized data. The core features of CTAB-GAN+ include as follows: (1) introduction of the auxiliary component, i.e., classifier or regressor, into conditional GAN, (2) effective data encodings for mixed and simple Guassian variables, (3) a novel construction of conditional vectors, and (4) tailored DP discriminator for tabular GAN. Results show that the synthetic data of CTAB-GAN+ results into higher ML utility and higher similarity against ten baselines on seven tabular datasets. The overall improvement on classification datasets is at least 56.4% (AUC) and 33.5% (accuracy) compared to related studies with no privacy guarantees. When turning on differential privacy, CTAB-GAN+ outperforms baselines at least 7.8% and 21.9% on F1-Score under privacy budgets ϵ = 1 and ϵ = 100. As future study, we plan to expand the range of data types that our algorithm can handle to better represent all types of data columns. We also intend to develop a tool for automated data type detection to make our algorithm more user-friendly.

## Author's note

This manuscript delves into the pivotal realm of data science, emphasizing the generation of synthetic data using advanced machine learning models, specifically Generative Adversarial Networks (GANs) for tabular data. In the contemporary big data ecosystem, there's an increasing need to generate high-quality synthetic data, which not only resembles the original but also ensures stringent privacy safeguards. Our research, centered around the introduction of CTAB-GAN+, contributes to this need by blending robust data synthesis, data utility preservation, and the incorporation of differential privacy. Given the journal's commitment to advancing the frontiers of data science and exploring the challenges and opportunities posed by big data, our work is distinctly aligned with its vision.

## Data availability statement

Publicly available datasets were analyzed in this study. This data can be found at: http://archive.ics.uci.edu/ml/datasets; https://www.kaggle.com/datasets/mlg-ulb/creditcardfraud; https://www.kaggle.com/datasets/itsmesunil/bank-loan-modelling; https://www.kaggle.com/datasets/mirichoi0218/insurance; https://www.kaggle.com/datasets/harlfoxem/housesalesprediction.

## Author contributions

ZZ: Conceptualization, Data curation, Formal analysis, Investigation, Methodology, Project administration, Software, Supervision, Validation, Visualization, Writing—original draft. AK: Data curation, Methodology, Software, Validation, Writing – original draft. RB: Conceptualization, Methodology, Supervision, Writing—review and editing. HV: Supervision, Writing—review and editing. LC: Conceptualization, Funding acquisition, Methodology, Resources, Supervision, Writing—review and editing.

## Footnotes

^1^ https://github.com/Team-TUD/CTAB-GAN-Plus-DP

^2^ http://archive.ics.uci.edu/ml/datasets

^3^ https://www.kaggle.com/mlg-ulb/creditcardfraud,itsmesunil/bank-loan-modelling

^4^ https://www.kaggle.com/mirichoi0218/insurance,harlfoxem/housesalesprediction

^5^ http://shakedzy.xyz/dython/modules/nominal/#compute_associations

^6^ https://github.com/BorealisAI/private-data-generation
